# Integrated analysis of online signals and insight generation about digital conversations on COVID-19 vaccines in Eastern and Southern Africa: a longitudinal analysis of social listening data

**DOI:** 10.1186/s12919-023-00261-2

**Published:** 2023-07-04

**Authors:** Silvia Sommariva, Helena Ballester Bon, Sofia De Almeida, Jenna Mote, Sijmen Brouwers, Massimiliano Sani, Natalie Fol

**Affiliations:** UNICEF Eastern and Southern Africa Regional Office, Social and Behaviour Change Section, Nairobi, Kenya

**Keywords:** COVID-19, Social listening, Social media, Digital engagement, Vaccine, Vaccine equity, Misinformation

## Abstract

**Background:**

During the COVID-19 pandemic, social listening programs across digital channels have become an integral part of health preparedness and response planning, allowing to capture and address questions, information needs, and misinformation shared by users. This study identifies key social listening trends around COVID-19 vaccines in Eastern and Southern Africa and analyses how online conversations about this issue evolved over time.

**Methods:**

A taxonomy developed and refined in collaboration with social and behaviour change teams was used to filter online conversations into nine subtopic categories. The taxonomy was applied to online content tracked in 21 countries in Eastern and Southern Africa over the period December 1, 2020—December 31, 2021. Metrics captured included volume of posts or articles and related user engagement. Qualitative analysis of content was conducted to identify key concerns, information voids and misinformation.

**Results:**

Over 300,000 articles and posts about COVID-19 vaccines shared by users or outlets geolocated in the region were analysed. These results generated over 14 million engagements on social media and digital platforms. The analysis shows how conversations about access and availability of vaccines represented the largest share of engagement over the course of the period. Conversations about vaccine effectiveness and safety represented the second and third largest share of engagement, with peaks observed in August and November 2021. Online interest in childhood vaccination increased over time as vaccine eligibility criteria expanded in some countries in the region. Conversations mentioning mandates and certificates peaked in the last quarter of 2021, as governments as private sector entities expanded vaccine requirements.

**Conclusions:**

Findings from this study show the importance of monitoring conversation trends over time and adjust social listening data collection systems to include emerging topics. The study also points to the need to consider concerns, information voids and misinformation around effectiveness and safety of vaccines in the context of overall concern for vaccine availability and access in Eastern and Southern Africa. This is fundamental to inform social and behaviour change strategies that promote vaccine demand effectively, without increasing public frustration over vaccine availability challenges and downplaying concerns around vaccine equity.

## Introduction

Analysing the layers of influence on immunization-related behaviours is a complex and necessary process to identify and course-correct strategies aimed to increase vaccine demand. Insights drawn from social listening activities, defined as the systematic monitoring and analysis of public conversations on selected online and offline channels, can contribute to shedding a light on how the *Journey to Health and Immunization* unfolds in a specific context, which barriers individuals encounter and how they can be engaged in solutions [1, 2].

The field of social listening for public health preparedness and response planning precedes the COVID-19 crisis [3, 4]; however, the pandemic has put a spotlight on the need for public health systems to be a proactive part of the conversation to address information voids, correct false information, capture questions and concerns [5, 6]. The COVID-19 vaccine rollout has taken place at the intersection of two challenging phenomena from a social listening perspective: the COVID-19 *infodemic*, in which digital communication and social media have facilitated the sharing of an unprecedented volume of information [5], and the long-standing digital presence of the anti-vaccine movement [7–9]. The challenges for vaccine demand promotion in this context have therefore been unprecedented [10].

To capture fast-evolving conversations and pick up on early signals of misinformation, social listening strategies have been designed to plan for frequent tracking and analysis of the data available, often on a weekly or even more frequent basis. These constant data monitoring efforts have led to a large volume of social listening data, which can be further leveraged to understand how discourse evolved over time, which trends persisted, and which new issues emerged.

In the African continent, COVID-19 vaccine demand generation efforts have been facing several challenges, ranging from misinformation affecting beliefs and knowledge, to barriers to access points of service, to after-service concerns [11–14]. Online social media and digital news monitoring tools, in conjunction with other social listening initiatives both on digital and ground-level channels, have been used to inform the response to the pandemic [1]. This study analyses online conversations about COVID-19 vaccines in 21 countries of Eastern and Southern Africa during a one-year period, using a taxonomy developed and refined in collaboration with social and behaviour change experts working in the field of vaccine acceptance and uptake. Inputs from digital social listening have been fed regularly into the regional Community Feedback Mechanism managed by the Interagency Technical Working Group in Eastern and Southern Africa; however, the work conducted by the authors in this article focuses on digital channels as part of the broader digital engagement portfolio of activities. The goal is to understand which vaccine subtopics have been most central to the public conversation on digital channels, how the focus has evolved over the course of the period considered, and how these insights informed the roll-out of COVID-19 vaccines in the region, both from a demand generation perspective and on the service delivery front, including efforts to improve the experience of care.

## Methods

### Study design and setting

This study adopted a mixed methods approach to the analysis of social listening data collected through monitoring of digital platforms in Eastern and Southern Africa between *December* 2020 and December 2021.

### Materials and data collection process

Data points from online public content about COVID-19 vaccines, including social media posts (content shared through a social media user profile, including text, images, videos, or a combination of these items) and news articles published on digital outlets, were collected from a wide range of digital channels relevant to the Eastern and Southern Africa region using the subscription-based social listening tool Talkwalker. Data collection was based on the social listening strategy developed and implemented by UNICEF in Eastern and Southern Africa, in collaboration with partners, for COVID-19 Risk Communication and Community Engagement (RCCE) [15]. This strategy was implemented in part using the support of the social listening tool mentioned, with a full-time analyst dedicated to the regional monitoring and the development of weekly social listening reports, as well as involvement of a broader team of social and behaviour change specialists. Data were stored automatically within the tool and in a secured summary Excel file downloaded every month. Platforms included in the monitoring were popular social media (Facebook, Twitter, Instagram), digital news, blogs and forums geolocated in one of the 21 countries in Eastern and Southern Africa. Search queries on Google were also reviewed on a weekly basis using Google Trends. Other social media platforms like TikTok or WhatsApp were not systematically included in the monitoring due to constraints in the functionalities of the social listening tool. Relevant posts and articles were identified using keyword-based Boolean search strings about COVID-19 vaccines, resulting from a review process among social and behaviour change experts working in the field of vaccine demand. The process consisted in an independent review by three social and behaviour change (SBC) immunization experts working in the region, edits were reviewed and incorporated by the social listening analyst, and the final taxonomy and related keywords was shared to the experts for review and approval.

### Data analysis

A COVID-19 vaccine taxonomy was developed and applied to filter online conversations tracked into subtopic categories using the social listening tool (Table 1). Subtopics were identified based on digital social listening activities conducted in the region over the course of the pandemic response, as well as questions and rumours identified in 2020. For each subtopic, keyword-based Boolean search strings were developed using the same consultation process mentioned above. Keywords included were in English, French, Portuguese, Swahili, and additional local languages depending on input provided by UNICEF country offices in the region. The resulting search strings were used to categorize conversations around COVID-19 vaccine into subtopics. The taxonomy was initially developed in February 2021, as the vaccine was starting to roll out in the region and was refined over the course of the following months. It was tested and validated by review of the top 100 posts/articles by engagement for the week in which the test was conducted in May 2021, yielding an overall precision rate of 74%, and edits to the search strings were made to improve retrieval precision rate. The taxonomy was used systematically starting from May 2021 for the weekly reports and applied retroactively for the analysis of data between December 2021 and May 2021. Metrics analysed by subtopic included volume of posts or articles and related engagement. Quantitative analysis of digital analytics was conducted using Excel and was used to identify key trends in vaccine-related conversations and potential information voids. Within each subtopic, a qualitative analysis of content was conducted by the social listening analyst on a weekly basis to identify specific concerns, questions and misinformation. Findings were triangulated with other data sources, including surveys, online search trends, internal briefs and partners’ reports, and used to produce weekly highlights and recommendations to inform vaccine promotion and delivery. Additionally, the repetition of a piece of news across social media platforms and digital news outlets was also taken into considerations to determine the importance of issues in the overall conversation. The weekly reports were drafted by the social listening analyst, including proposed recommendations, which would be then discussed with two SBC Immunization specialists. The final reports were shared each week with SBC and Expanded Programme on Immunization (EPI) specialists in the region.

**Table 1:** Social listening taxonomy for COVID-19 vaccine conversations

Subcategory	Description
Effectiveness	Content discussing how well vaccines are working in the real world. Example keywords: effective*, success*, immun*
Safety	Content discussing risks associated with vaccination. Example keywords: AEFI*, unsafe, side effect*
Children	Content mentioning children as a target group for the vaccine. Example keywords: child*, infant, teen*
Certificates	Content mentioning vaccine-related documentation. Example keywords: certificate*, passport*, proof
Clinical trials	Content mentioning research studies that evaluated or are evaluating the effects of the vaccine on health outcomes. Example keywords: trial*, study, published
Fertility	Content mentioning concerns on the potential impact of the vaccine on fertility. Example keyword: sterile, pregnan*, menstrua*
Expiration	Content discussing vaccine expiration. Example keywords: shelf life, expired, expiration
Second dose/ Booster	Content discussing the second dose (for vaccines requiring two doses) or boosters. Example keywords: booster*, second, third
Availability and access	Content discussing accessing the vaccine or availability issues. Example keywords: eligib*, administration, equity, donation*, access*, vaccination center*

^*^used for multiple character searching

## Results

### Sample description

Over 300,000 articles and posts about COVID-19 vaccines shared by users or outlets geolocated in the region were tracked during the period December 2020-December 2021 in this sample. About half of the results tracked were from South African users or outlets, followed by 16% of content geolocated in Kenya, 8% in Uganda and 5% in Zimbabwe (Table 2). This distribution is partly a reflection of Internet penetration rates, which vary significantly across countries in the region: for example, according to World Bank data [16], 70% of the population uses the Internet in South Africa, compared to 1% in Eritrea. About 85% of the content was in English language, followed by Swahili (5%), French (2%), Portuguese (2%), Afrikaans (2%), Amharic (1%) and other languages (3%). Data about user gender were retrievable for 120,000 posts, with a breakdown 62% male – 38% female.

**Table 2:** Share of results by country, internet penetration and population statistics

	Share of sample results by country of origin^a^ (%)	Population using the Internet (%) *	Share of ESAR population (%) **
Angola	< 1	36	6
Botswana	4	64	0
Burundi	< 1	9	2
Comoros	< 1	8	0
Eritrea	< 1	1	1
Eswatini	< 1	30	0
Ethiopia	1	17	21
Kenya	16	30	10
Lesotho	< 1	43	0
Madagascar	< 1	15	5
Malawi	< 1	10	4
Mozambique	< 1	17	6
Namibia	2	41	0
Rwanda	1	24	2
Somalia	< 1	2	3
South Africa	54	70	11
South Sudan	< 1	7	2
Tanzania	3	22	11
Uganda	8	6	8
Zambia	< 1	20	3
Zimbabwe	5	29	3

^a^Geolocation of user or outlet account

^*^Internet penetration rates (population using the internet) are from World Bank, https://data.worldbank.org/indicator/IT.NET.USER.ZS, latest available data for each country

^**^Population data are expressed as percentage of total population in the region and are for 2023 based on the latest United Nations Population Division

**Fig. 1:**
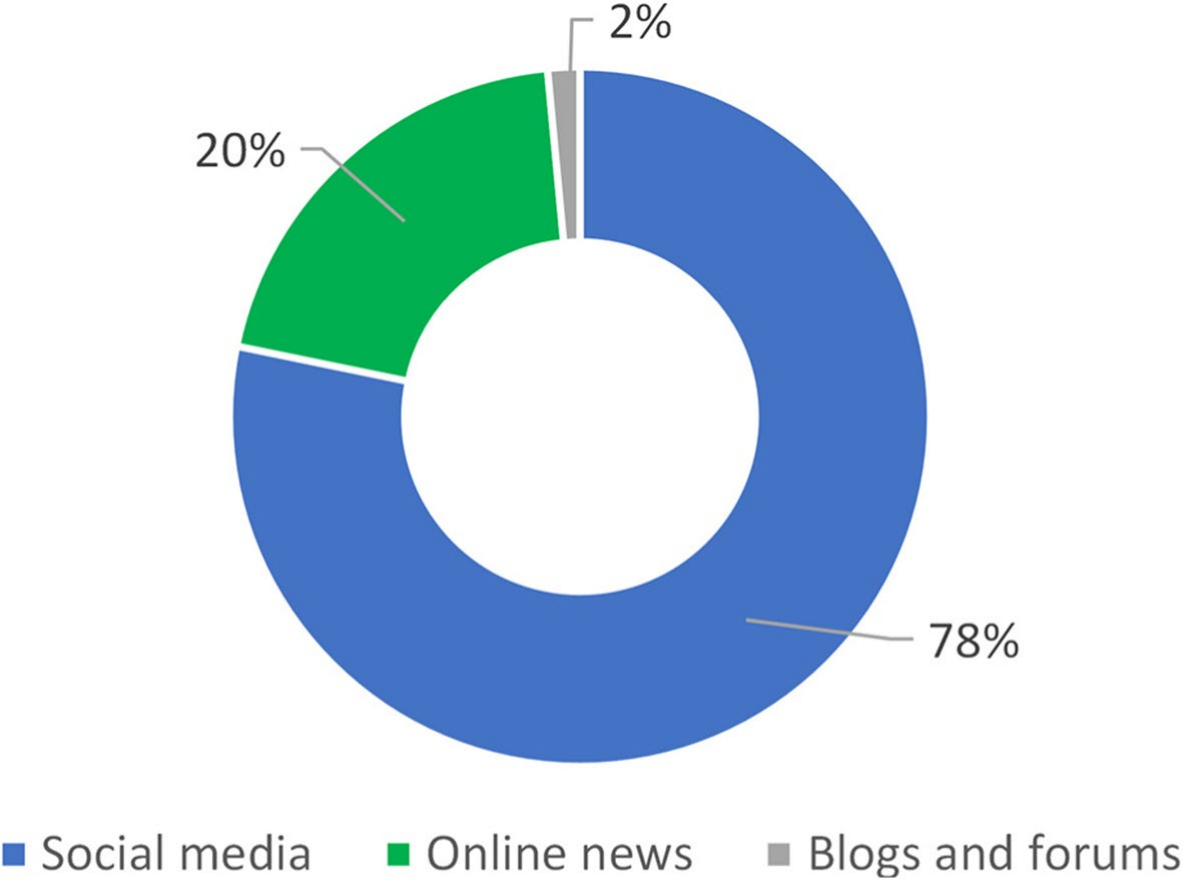
Share of engagements by channel

These results generated over 14 million engagements (likes/reactions, share, or comments) on social media and digital platforms. Seventy-eight percent of engagements were generated by content on social media, 20% by digital news articles and 2% by posts on blogs and forums (Fig. 1).

### Subtopics

**Fig. 2:**
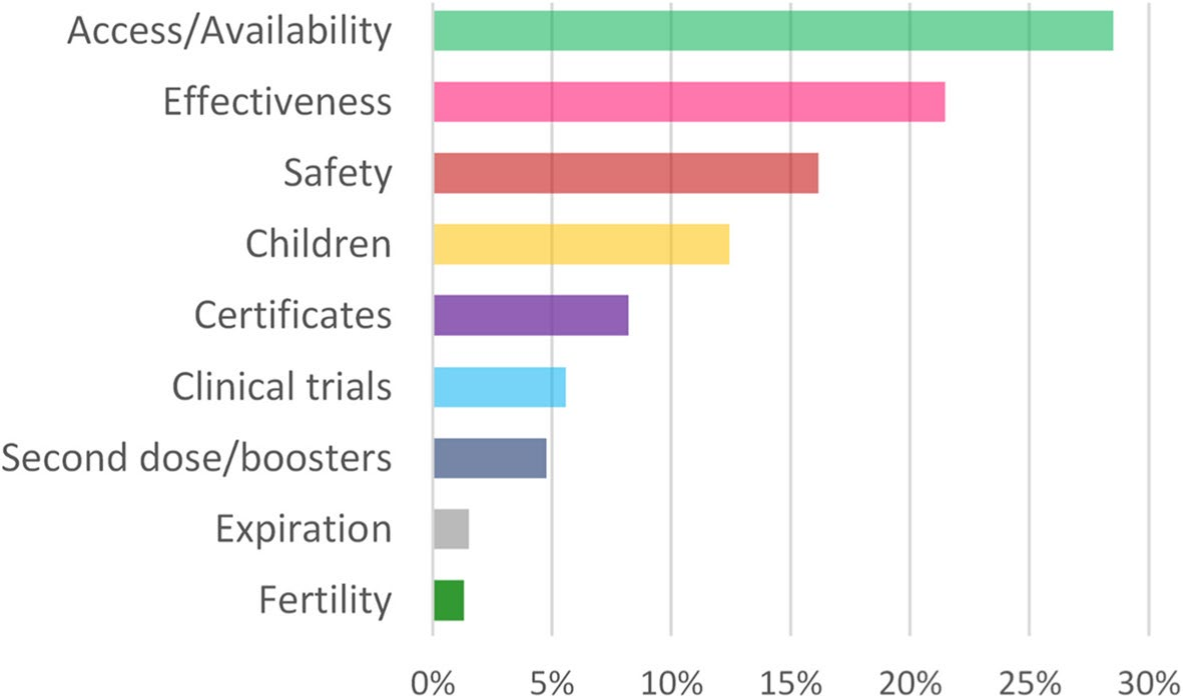
Share of engagement by subtopic

The analysis (see Fig. 2) shows how conversations about vaccine access and availability, which included content mentioning where the vaccine was administered, announcements of vaccine donations, and experiences of access challenges, represented the largest share of engagement over the course of the period (28%). Conversations about vaccine effectiveness and safety represented the second and third largest share of engagement (21% and 16% respectively).

**Fig. 3:**
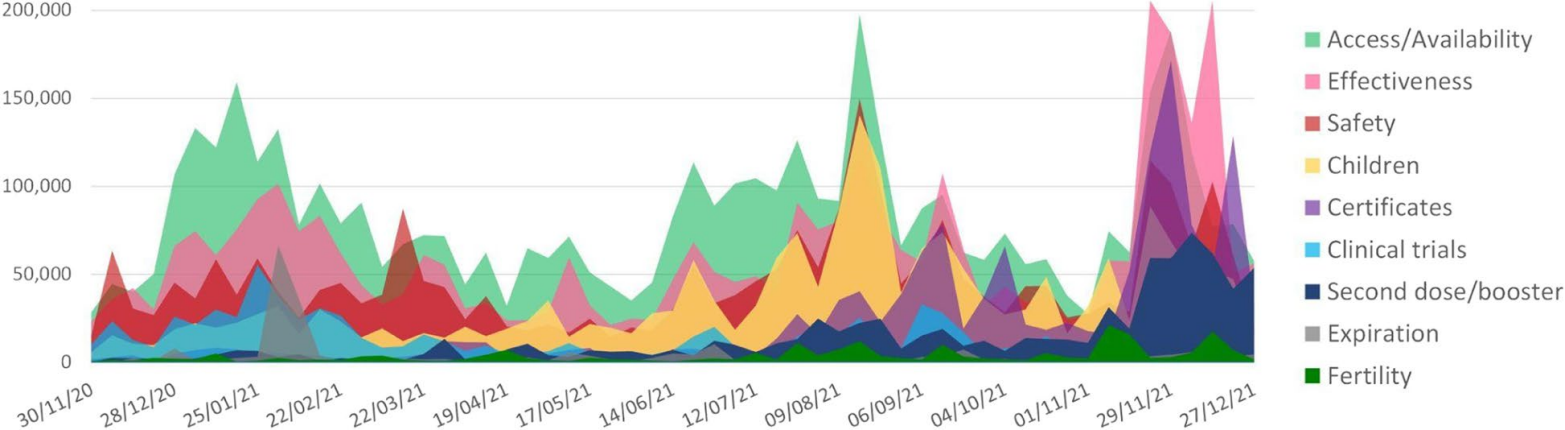
Engagement over time by subtopic

Peaks in user engagement about access and availability were tracked in December 2020-January 2021, and through the period June–August 2021 (Fig. 3). In January 2021, conversations about this subtopic were in large part driven by the news of a fire in a vaccine manufacturing plant in India, which generated concerns about production capacity [17]. An announcement by South Africa’s President reassuring the public that enough vaccines would be made available also generated interest [18], as did a statement by the World Health Organization inviting the population not to panic because everyone willing to get vaccinated would have the opportunity to do so [19]. News that U.S. President Biden intended to join COVAX also generated high engagement [20]. In June–August 2021, interest in vaccine access and availability increased in conjunction with a wave of COVID-19 cases in some of the countries in the region including South Africa, Kenya, Uganda and Zimbabwe. During this time, users were calling for wider vaccine access, particularly for healthcare workers. Search engine queries on how to access services were also tracked in several countries throughout the period.

While “access and availability” was the topic that generated most engagement overall and for most of the period considered, conversations around effectiveness represented the largest share of engagement during December 2021. This peak occurred after the World Health Organization declared the COVID-19 variant B.1.1.529 (Omicron) a variant of concern [21]. The news generated questions about the effectiveness of vaccines against infection with the new variant, as well as intensified the promotion of vaccine uptake to limit the impact on health outcomes and lower the chances of virus mutations. Conversations about vaccine certificates during this period were related to mandates announced or being discussed in several countries in the region, including South Africa, Kenya, Lesotho, and Malawi. Reports of fake certificates being sold were also circulating.

The level of interest around vaccine safety remained rather consistent over time, usually representing the third or fourth category of engagement. In August 2021, a video featuring a prominent South African surgeon saying that vaccines are unsafe generated high interest [22]. Concerns about side effects were also tracked, with users reporting their own experience or amplifying reports of Adverse Events Following Immunization (AEFIs). Online interest in childhood vaccination increased over time as vaccine eligibility criteria expanded in some countries in the region, with a peak in August 2021, when discussions around future vaccination of people below 18 were starting in some countries. During this time, some active anti-vaccine accounts in the region started to focus their content on discouraging child vaccination. Content around clinical trials was more prominent at the beginning of 2021, when Uganda announced the launch of a clinical study for a locally developed vaccine and South Africa struggled to secure doses despite participating in vaccine trials in 2020 [23]. Interest in the administration of second doses and booster shots peaked towards the end of the period, when concerns around the spread of the Omicron variant drove public health officials to encourage healthcare workers in South Africa vaccinated with the Johnson & Johnson jab to get a booster dose [24]. Interest in vaccine expiration saw relatively higher volume in February 2021, when it was found that many of the doses received in South Africa were set to expire in April. Conversations about COVID-19 vaccines and fertility were continuously monitored over the course of the period. Several of the articles and posts identified encouraged pregnant women to get the jab. Concerns tracked included worries about the impact of the vaccine on menstrual periods, and questions on whether the vaccine could cause sterility in men and women.

## Discussion

Application of a COVID-19 vaccine taxonomy to filter online content geolocated in Eastern and Southern Africa helped social and behaviour change analysts working on the vaccine rollout to triangulate qualitative insights into the concerns and questions of target populations with a quantitative snapshot of which subtopics were generating most engagement at a given time. This process has allowed to focus the development of operational recommendations on the key subtopics of concern, as well as to monitor the evolution of conversations over time.

For example, ongoing monitoring of COVID-19 vaccine conversations in the region showed that concerns about equitable access to services and disruptions due to the pandemic were persistent during the period considered. Availability and access challenges were of particular concern as more countries started to implement vaccination requirements at the end of 2021 and into 2022, for example by linking access to public services to full immunization or mandating vaccination as a pre-requisite for employment. In some cases, the tracking of questions from online users on how/where to access the vaccine prompted the identification of information voids, due to the lack of updated, easy to find (e.g. not showing up on search engines) and clear information. While access has been expanded thanks to the relentless work of COVID-19 response stakeholders in the region, including governmental institutions at different levels, community-based groups and international organizations, analysis of online conversations shows that unequal allocation of vaccines globally and logistical access barriers remained a key concern during the assessed period. This social listening insight, shared with EPI and social and behavior change programmes, has guided the development of regional recommendations for action throughout the course of 2021, prioritizing calls to advocate for vaccine equity across regions and within countries, and to not discount logistical barriers to access and continue to provide clear and localized information on eligibility criteria, vaccine registration and services location. The analysis also guided recommendations around the tone of vaccine demand campaigns, to acknowledge potential frustration due to access challenges and manage expectations about when vaccines would become available for different population groups.

Another instance in which social listening insights were able to provide in-depth understanding of and quickly address concerns has been related to AEFIs. For example, in May 2021, monitoring picked up on early signals of online conversations around a suspected adverse event in Madagascar. A local university professor had died while jogging the day after receiving the COVID-19 vaccine; social media users on Facebook and local blogs shared the news, saying the vaccine was unsafe. Due to the evolving nature of the issue, several data voids were identified such as information on whether people can practice sports after getting the vaccine or whether a healthcare check-up is necessary to safely receive the vaccine (there were rumours that everyone needed to undergo a health check before receiving the shot). This triggered the activation of risk communication plans at country level, with international partners supporting the government in drafting messages (including press releases and a Q&A document to disseminate through the COVID-19 hotline) to manage concerns and rumours while an official investigation to assess causality was ongoing.

Social listening was also instrumental to understand concerns of specific population groups. For instance, insights on online conversations regarding COVID-19 vaccines and pregnancy helped inform the development of social media content addressing concerns tracked, for example that the vaccine causes miscarriages or can impact the health of the expectant mother, that the vaccine has not been sufficiently tested for safety during pregnancy, and that side effects can be passed to babies through breastfeeding. The analysis of conversations identified that users were interested in other people’s experiences, prompting a recommendation to prioritize human interest stories of mothers who received the vaccine while pregnant and were able to safely deliver their babies. It also showed that users were eager to engage with healthcare workers in safe spaces to ask questions; this and other data on trust in different spokespersons suggested that the social media content should also feature healthcare staff willing to engage in open conversations about the issue.

The taxonomy used in this study has been developed in a short period of time in support of the analysis of social listening insights within the larger efforts of COVID-19 response in the region, thus limiting the ability to further review and test the selected subcategories. Subcategories were identified with the objective to inform the vaccine rollout in a specific geographical area and moment in time and were not meant to provide a comprehensive representation of all issues that surround vaccine programming. Additional limitations of the analysis are related to the sample of articles and posts selected, which is influenced by accessibility of online content through the social listening tool employed. To this end, it is not possible to assess whether the sample of results is representative of overall conversations and to which extent conversations in different languages had different characteristics. Moreover, the analysis does not account for content not publicly available such as messages circulating on closed social media groups and messaging apps. It is also important to note that metrics of volume and engagement of posts circulating on social media do not equate with importance of issues as perceived by community members. A regional approach to the analysis of engagement tends to give more prominence to countries and areas with larger populations, higher internet penetration and more pervasive social media use. This can lead to miss relevant conversations happening in smaller countries and in rural contexts. As illustrated in Table 2, internet penetration varies considerably across countries in Eastern and Southern Africa, and data from the sample are in part a function of the level of internet use in different contexts. To this end, integration with offline mechanisms, not considered in this study, is fundamental to enhance relevance of social listening findings. Language-related limitations also apply, in part because keyword search strings included a higher percentage of English terms. Due to limitations of AI-informed sentiment algorithms in social listening tools, the analysis does not include data on the tone of conversations tracked, especially as the results are not always reliable for non-English language content. User gender data were available only for one social media channel and could therefore not be used to further segment the findings.

Overall, the analysis and observations that followed contribute to understand the complex role and potential of social listening in support of vaccine demand efforts and in a context of emergencies like COVID-19. First, the employment of a social listening taxonomy on online conversations provides a big picture view of which issues are drawing most interest among users, evidence that can be used as a jump off point to dive deeper into specific pieces of content, such as articles or posts that generated high engagement or that appeared to be circulating on multiple platforms. This approach can be particularly helpful to prioritize areas of investigation and action, which is essential in resource- or time-constrained settings. Second, digital social listening can help identify early signals of concerns and data voids in quickly evolving situations such as during AEFI’s investigations; this can inform the development of content that aims to increase availability of (and hopefully user exposure to) accurate information. Third, social listening can provide valuable recommendations also on how certain messages should be delivered, both in terms of timing (e.g. if there is frustration due to lack of vaccine availability, pushing demand messages could backfire) and in terms of spokespersons and trusted messengers.

## Conclusions

Results from this analysis of social listening data show that designing interventions that address logistical barriers and improve the convenience of services is important to ensure sustained demand and uptake of vaccines. Tracking of concerns related to safety, particularly users reporting side effects following immunization, also points to the need for increased communication around after service support, and raising awareness around vaccine surveillance processes within countries. Future research could benefit from access to disaggregated social listening data, particularly to conduct segmentation of results by gender, age and rural/urban context. Overall, findings from this study show the need to consider concerns, information voids and misinformation around effectiveness and safety of vaccines in a context in which access and availability was the most prevalent concern. This is fundamental to inform communication strategies that promote vaccine demand effectively by addressing rumours and providing accurate information, without increasing public frustration over vaccine availability challenges and downplaying concerns for vaccine equity.

## Data Availability

The datasets analysed during the current study are available from the corresponding author on request.

## Abbreviations

AEFIs: Adverse Events Following Immunization
RCCE: Risk Communication and Community Engagement
UNICEF: United Nations Children's Fund
